# Signaling pathways and molecular mechanisms of lupeol in colorectal cancer: A comprehensive review

**DOI:** 10.1016/j.jaim.2025.101270

**Published:** 2026-01-29

**Authors:** Mohd Asad Farooqi, Kantrol Kumar Sahu

**Affiliations:** AB-04, Institute of Pharmaceutical Research, GLA University, Mathura, UP, 281406, India

**Keywords:** Colorectal cancer, NF-κB, COX-2/PGE2, IL-6/STAT3, Lupeol, Bioavailability, Anticancer, Signaling pathways

## Abstract

Colorectal cancer (CRC) is a serious worldwide health concern associated with chronic inflammation and dysregulation of numerous signaling pathways, including IL-6/STAT3, NF-κB, COX-2/PGE2, and IL-23/Th17. These pathways aid in tumour development and progression by increasing the manufacture of inflammatory mediators, anti-apoptotic gene expression, cell proliferation, and angiogenesis. Lupeol, a naturally occurring lupine-type pentacyclic triterpenoid, has sparked widespread interest due to its several pharmacological qualities, which include antioxidant, anti-inflammatory, anticancer, and antibacterial capabilities. Despite its enormous medicinal promise, low water solubility and bioavailability have hampered its clinical use. Recent advances in nano-based delivery systems and the invention of lupeol derivatives have increased its bioavailability and bioactivity, making it a promising candidate for CRC treatment. This study seeks to inspire additional research into lupeol's significance as a nutraceutical intervention in CRC by integrating current knowledge and investigating novel techniques to increase its clinical efficacy.

## Highlights

• Lupeol, a pentacyclic triterpenoid, exhibits potent anticancer, anti-inflammatory, and antioxidant properties, making it a promising candidate for colorectal cancer (CRC) treatment.
• Colorectal cancer progression is linked to dysregulated signalling pathways such as NF-κB, IL-6/STAT3, COX-2/PGE2, and IL-23/Th17, which lupeol effectively modulates.
• Lupeol induces apoptosis in CRC cells by influencing mitochondrial pathways, suppressing tumour growth, and inhibiting cell proliferation and angiogenesis.
• Challenges such as low water solubility and bioavailability have hindered clinical applications, but advancements in nano-based delivery systems have improved its therapeutic potential.
• Further research, including preclinical and clinical studies, is essential to validate lupeol's efficacy as a nutraceutical intervention for colorectal cancer treatment.

## 1 Introduction

**Fig. 1:**
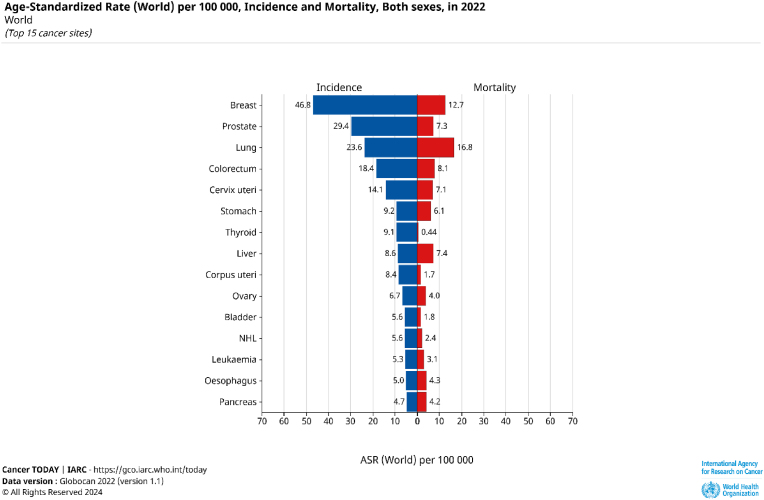
According to estimates of the age-standardized rate, incidence, and death for cancer in both genders in 2022 (data source: GLOBO-CAN 2022; map production: IARC, http://gco.iarc.fr/today, retrieved on December 01, 2024).

Colorectal cancer (CRC) is a severe universal health worry, as it is the third most prevalent disease diagnosed worldwide and the second-largest contributor to cancer-related deaths in 2022 (Fig. 1) [1]. Colorectal cancer caused an estimated 1.9 million new cases and 935,000 deaths in 2020. The occurrence of CRC is expected to rise sharply, with projections suggesting over 3.2 million new cases annually by 2040, fueled by population growth, ageing, and the widespread adoption of Westernized lifestyles in developing regions. High-income countries historically report greater CRC incidence, but the burden is increasing speedily in low- and middle-income nations due to dietary changes, sedentary behaviours, and rising obesity rates[[1], [2], [3]] Genetic, environmental, and routine factors are the causes of colorectal cancer (CRC). Heavy Diets in red and processed meats, smoking, extreme alcohol use, sedentary lifestyles, and chronic illnesses, including inflammatory bowel disease, are known risk factors. Despite being preventable through lifestyle changes and early detection, CRC mortality remains high due to challenges in achieving widespread access to screening and treatment in resource-limited settings [4,5]. Screening programs in high-income nations have substantially reduced CRC mortality by permitting early detection and eliminating precancerous lesions. However, significant gaps in implementation persist in many parts of the world, emphasising the need for equitable healthcare access. Emerging research highlights the promise of innovative therapeutic approaches, including immunotherapies, personalized medicine, and natural product-based treatments. These strategies offer the potential to address unmet clinical needs, particularly in overcoming treatment resistance and improving patient outcomes. As the global burden of CRC grows, a multi-faceted approach encompassing prevention, early detection, and advanced therapeutics is crucial to mitigate its impact and improve survival outcomes [5,6].

Recent therapeutic strategies, including chemotherapy, surgery, radiotherapy, and targeted therapy, have substantially improved CRC management. However, these approaches face limitations such as significant side effects, high costs, and the appearance of drug resistance. For instance, the widespread use of chemotherapeutics like 5-fluorouracil and oxaliplatin has improved survival rates but often leads to severe toxicities and relapse in advanced cases. Additionally, targeted therapies, while promising, are not universally effective due to interindividual variability in molecular tumour profiles [7,8].

The need for novel therapeutic agents is critical to address these gaps. Natural products, particularly phytochemicals such as lupeol, have garnered attention due to their multifaceted mode of action, including anti-inflammatory, pro-apoptotic, and anti-angiogenic properties and attractive physicochemical properties (Table 1). Lupeol, a pentacyclic triterpenoid originating in a diversity of medicinal plants, has shown significant promise in preclinical models of CRC by modulating pathways complex in cancer progression, for example, PI3K/AKT/mTOR and NF-κB. These attributes position it as a potential adjunct or alternative to current CRC therapies, warranting further research and clinical validation [9,10].

**Table 1:** Physicochemical properties of lupeol.

Property	Description	Reference
Molecular Formula	C30H50O	[52]
Density	Approximately 0.9 g/cm^3^ (typical for triterpenes).	
Crystal Structure	Orthorhombic crystal system, forming needle-like or prismatic crystals when purified.	
Molecular Weight	426.72 g/mol	[48]
Boiling Point	It is not typically applicable due to degradation at high temperatures; decomposition occurs before boiling.	
Log P (Partition Coefficient)	>5 (high lipophilicity), indicating good membrane permeability and retention in lipid-rich environments.	[32]
Polarity	Nonpolar compound, which contributes to its lipophilic behaviour and biological membrane interaction.	
Melting Point	215°C-216 °C	[53]
Solubility in Water	Practically insoluble in water (<0.1 mg/mL) due to its nonpolar nature.	
Thermal Stability	Stable under normal laboratory conditions but susceptible to degradation at high temperatures or when exposed to oxidative environments.	

This integrative understanding highlights the urgent need for innovative, low-toxicity treatments that leverage natural compounds like lupeol to complement or replace existing CRC interventions.

## 2 Molecular mechanisms implicated in colorectal cancer

The active growth of colorectal cancer has been associated with several immune system signalling pathways, such as NF-κB, PGE2/COX-2, IL-6/STAT3, and IL-23/Th17. Additionally, the gut microbiome influences the onset and course of colorectal cancer. Signals from chronic Inflammation may boost angiogenesis, promote the development of epithelial cells, and enhance oxidative stress, all of which may lead to cancer. [11,12]. Below is a summary of the immunological reactions, signalling pathways, and other elements that subsidise the pathophysiology of colorectal cancer carcinogenesis following colon inflammation.

### 2.1 NF-κB mechanism

**Fig. 2:**
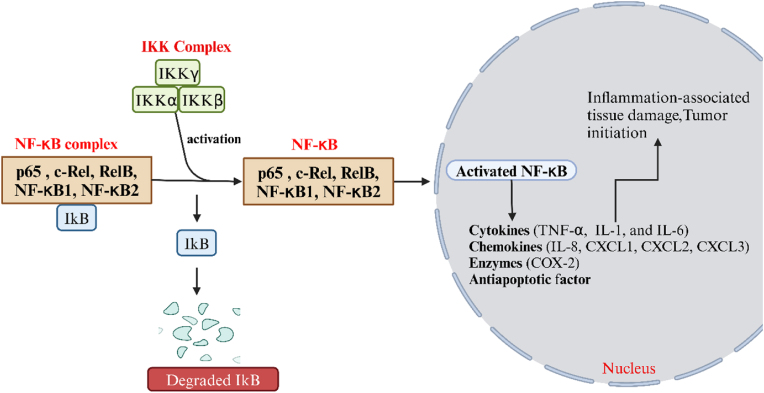
NF-κB complexes are localised in the cytoplasm when an NF-κB inhibitor is activated. Enzymes like COX-2, cytokines (such as IL-1, IL-6, and TNF-α), and chemokines (such as CXCL1, CXCL2, CXCL3, and IL-8) are all produced when NF-κB is active, and these can damage tissues and promote the formation of tumours.; IKK, IκB kinase; IKKα,IκB kinase alpha; IKKβ, IκB kinase beta; IKKγ, IκB kinase gamma; NF-κB, Nuclear factor kappa-light-chain-enhancer of activated B cells; IκB, Inhibitor of kappa B; TNF-α,Tumor necrosis factor-alpha; IL-1,Interleukin-1; IL-6, Interleukin-6; IL-8, Interleukin-8; CXCL1,C-X-C motif chemokine ligand 1; CXCL2, C-X-C motif chemokine ligand 2; CXCL3, C-X-C motif chemokine ligand 3; COX-2,Cyclooxygenase-2.

Nuclear Factor-Kappa B proteins, or NF-κB proteins, are vital in developing colorectal cancer. Numerous physiological processes, such as immunological reactions, cell death, cell division, cell cycle progression, inflammatory conditions, and malignant transformation, are regulated by NF-κB transcription factors. The five members of the regulatory complex are c-Rel, p50/p105 (NF-κB1), p65 (RelA), RelB, and p52/p100 (NF-κB2) [13] In most cells, the cytoplasmic localisation of NF-κB complexes is regulated by NF-κB (IκB) protein inhibitors. IκB kinase (IKK) stimulates IκB, which in turn stimulates NF-κB. IKK is a protein complex that contains the catalytic subunits IKKα and IKKβ in addition to a regulatory component called NF-κB essential modulator (NEMO) or IKKγ [14]. IKK/NF-κB pathway stimulation is a key process for cell endurance in various kinds of cancer. The deregulating of usual NF-κB action, whether by expression of an aberrant protein variant or interference through the transcriptional machinery beginning as of the typical gene, has been shown to contribute to the development of solid tumours, leukaemia, and lymphomas [15]. Moreover, the initiation of apoptosis-resistant genes by members of the NF-κB family also leads to the expansion of tumours, which results in resistance to chemotherapy and radiation. NF-κB regulates many cytokines and influences the inflammatory progressions unique to IBD [16]. According to research, almost 50 % of those with CRC and cancers linked to colitis had aberrantly active NF-κB. The participation of NF-κB in the growth of colorectal cancer has been validated by mouse experiments. Boost levels of NF-κB proteins in macrophages and epithelial cells are seen in UC patients, suggesting that NF-κB is constantly active. Numerous proinflammatory triggers, including viruses, bacteria, proinflammatory cytokines, and substances that damage DNA, can activate NF-κB. It has been demonstrated that experimental colitis can be effectively reduced by blocking such signal transmission [17]. The increased production of cytokines (IL-1, IL-6 & TNF-α), chemokines (CXCL1, CXCL2, CXCL3, and IL-8, among others), and enzymes (COX-2) result from the translocation of active NF-κB into the nucleus. Tumor growth and tissue damage are caused by these variables (Fig. 2) [18,19]. Increased IKK/NF-κB activity can promote cell survival by causing aberrant overexpression of adhesion proteins, chemokines, tumorigenic proteins, and apoptosis inhibitors. Furthermore, the NF-κB pathway has been revealed to contain genes that encode antiapoptotic regulators, such as the B-cell lymphoma 2 (BCL-2) associated protein (BFL1), the growth arrest and DNA damage-inducible 45β (GADD45β), and the B-cell lymphoma XL (BCL-XL) [20]. It is widely acknowledged that activating these antiapoptotic genes ensures that malignant cells will continue to exist and spread. Therefore, by sustaining a chronic inflammatory progression within the digestive tract's mucosal lining, NF-κB may be vital to the growth of colorectal cancer.

### 2.2 IL-6/STAT3 mechanism

**Fig. 3:**
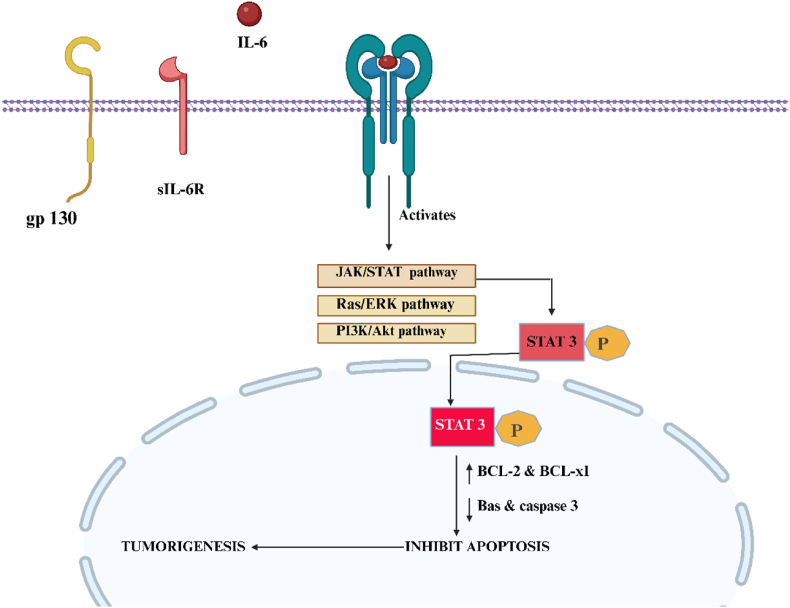
The interplay between IL-6 and the soluble IL-6 receptor (sIL-6R) initiates gp130 homodimerisation, which in turn triggers the activation of various pathways, including PI3K/Akt, Ras/ERK, and JAK/STAT. JAK phosphorylates gp130, leading to the formation of docking sites for STAT3. Subsequently, phosphorylated STAT3 migrates to the nucleus, where it inhibits pro-apoptotic proteins like Bax and Caspase-3 while enhancing the expression of anti-apoptotic proteins like BCL-2 and Bcl-xL. This process ultimately hinders apoptosis and supports tumour cell survival.; IL-6, Interleukin-6; sIL-6R, Soluble interleukin-6 receptor; gp130, Glycoprotein 130; JAK/STAT, Janus kinase/Signal transducer and activator of transcription; Ras/ERK, Ras/Extracellular signal-regulated kinase; PI3K/Akt, Phosphoinositide 3-kinase/Protein kinase B; STAT3, Signal transducer and activator of transcription 3; BCL-2, B-cell lymphoma 2; BCL-xL, B-cell lymphoma-extra-large; Bax, Bcl-2-associated X protein; Caspase 3, Cysteine-aspartic protease 3.

In UC-CRC, NF-κBs play a crucial role in regulating IL-6, a cytokine known for its pro-inflammatory and protumor properties. Individuals with IBD have significantly raised IL-6 levels within their intestinal lining and serum, which correlates favourably with the harshness of the inflammation [21]. The relation between soluble IL-6 & the soluble IL-6 receptor (sIL-6R) recruits and homo dimerises two gp130 subunits, which in turn triggers intracellular signalling pathways like the Ras/ERK(Extracellular Signal-regulated Kinase), JAK (Janus kinase)/STAT, and PI3K (Phosphoinositide 3-kinase)/Akt pathways [22,23]. When JAK is activated, several tyrosine residues in the cytoplasmic tail of gp130 become phosphorylated, creating docking spots for STAT3 [24]. Once phosphorylated, STAT3 may reach the cellular nucleus and prevent apoptosis by raising the production of antiapoptotic proteins, including BCL-2 and Bcl-xl and downregulating the expression of Bax and Caspase 3 [15]. The apoptotic resistance of CD4+T cells promotes tumorigenesis and subsidises the long-term maintenance of gastrointestinal inflammation (Fig. 3) [25]. IL-6 is well recognised as a major promoter of tumour growth in the background of colitis-related cancer, and STAT3 is crucial for transmitting IL-6-induced signals that promote tumour growth [24,26].

### 2.3 IL-23/Th17 mechanism

**Fig. 4:**
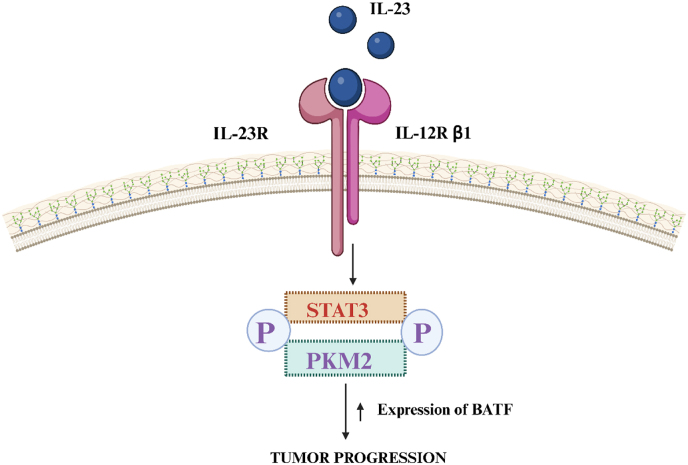
Activation of IL-23R and associated signalling pathways, such as the phosphorylation of PKM2 and STAT3, led to the development of tumours.; IL-23, Interleukin-23; IL-23R, Interleukin-23 Receptor; IL-12R β1, Interleukin-12 Receptor Beta 1; STAT3, Signal Transducer and Activator of Transcription 3; PKM2, Pyruvate Kinase M2; BATF, Basic Leucine Zipper ATF-Like Transcription Factor.

The conversion of UC to CRC is significantly unfair by the IL-23/Th17 path. [27]. The cytokine IL-23 is a heterodimer comprising the p19 & p40 subunits. The p40 subunit binds to the β1 chain of the IL-12 receptor (IL-12R), while the p19 attaches itself to the IL-23 receptor (IL-23R) by its N-terminal immunoglobulin site, rebuilding the p19 helical domain [19]. The beginning of downstream signalling pathways occupied in inflammation and the advancement of cancer depends on the relationship between IL-23 and its receptor. Through the initiation of IL-23R and subsequent signalling actions, such as the phosphorylation of STAT3 and PKM2, the IL-23/Th17 pathway plays a crucial part in the conversion of UC into CRC. [28]. The development of colitis-related tumours and the advancement of spontaneous colon tumours result from increased Batf transcription factor expression in CRC tissues (Fig. 4). The stimulation of IL-17 can cause Th17 cells to differentiate into Th1 cells, which consequently promotes the synthesis of mucosal innate IL-12 and IL-23, adding even more to the pathophysiology of colitis. Together with Th17 cytokines, IL-23 is essential for preserving intestinal homeostasis and protection. It also regulates T-cell-independent mechanisms of gastrointestinal inflammation [29,30].

### 2.4 PGE2/COX-2 mechanism

**Fig. 5:**
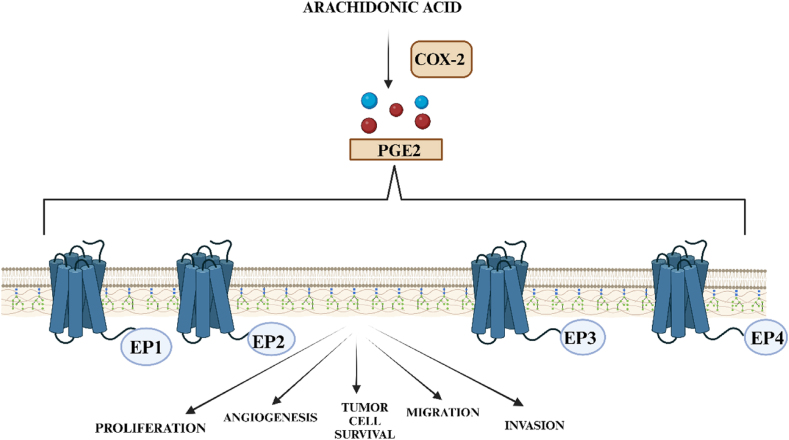
Prostaglandin E2, or PGE2, is formed via the enzyme COX-2 in response to inflammation. PGE2 interacts with the E-type prostanoid (EP) receptors upon immune cells. Through pleiotropic activity, PGE2 can stimulate colorectal cancer angiogenesis, invasion, migration, and proliferation.COX-2 – Cyclooxygenase-2; PGE2, Prostaglandin E2; EP1, Prostaglandin E receptor 1; EP2, Prostaglandin E receptor 2; EP3, Prostaglandin E receptor 3; EP4, Prostaglandin E receptor 4.

A crucial participant in colonic irrigation inflammation and carcinogenesis is COX-2. About 85 % of colorectal cancers have been found to have elevated COX-2 expression, which is associated with a worse prognosis. Additionally, COX-2 overexpression has been seen in neoplastic tissues linked to colitis in IBD patients as well as in patients experiencing active inflammation. Prostaglandin E2 (PGE2), which interrelates with immune cell E-type prostanoid (EP) receptors, is produced by the enzyme COX-2 in response to inflammation. In particular, CRC is often associated with an upregulation of the EP4 receptor. One way that COX-2 encourages the growth of tumours is by the production of antiapoptotic proteins like BCL-2. This leads to resistance to apoptosis. Moreover, elevated levels of matrix metalloproteinase (MMPs) and cancerous cells' migration ability are associated with COX-2 overexpression. [30]. When COX-2 is raised, PGE 2, which promotes proliferation, existence, angiogenesis, migration, and invasion in colorectal tumours, is increased (Fig. 5) [13].

### 2.5 Purinergic signalling

According to some reports, the gut immune system's balance is preserved via the purinergic pathways. ATP (P2X) and adenosine (P1) receptors are among the receptors involved in purinergic signalling. This signalling pathway influences cell division, proliferation, and death, in addition to immunological responses and inflammation. Through proinflammatory microenvironments and mutations brought on by oxidative stress, long-lasting intestinal inflammation, as shown in UC, aids in the start and spread of tumours. [13,31]. IBD patients had higher levels of mononuclear cells in the lamina propria and P2X7 receptors in the layer of epithelial cells. Research employing mice lacking the P2X7 receptor has demonstrated a drop in the build-up of regulatory T cells (Tregs) in the colon as well as a drop in inflammatory cytokines. [31]. Mice preserved using dextran sulfate sodium (DSS) and azoxymethane (AOM) showed increased tumour occurrence when the P2X7 receptor was inhibited, despite its anti-inflammatory effects. In this specific mouse model, the impact of tumour development was related to an increase in TGFβ1 expression, which directly excites the proliferation of epithelial cells and influences the immunosuppressive microenvironment [32,33].

### 2.6 APC, KRAS, and TP53 mutation

Mutations in genes similar to oncogenes (KrAs), tumour protein 53 (TP53), and Adenomatous polyposis coli (APC) [34]. It also results in changes that are important in the onset and course of colorectal cancer (CRC) linked to ulcerative colitis. Many mutations in the TP53 gene, which inhibits the growth of tumours, are detected in CRC samples. Furthermore, the brain and gastrointestinal tract are among the tissues that express APC, a big protein that serves as structural support. Within the Wnt signalling pathway, it plays a critical role in regulating the oncogenic protein beta-catenin. KrAs are described by way of a “toggle” in the signals sent by cancer cells that control how malignant their actions are. A defective p53 protein caused by Tp53 mutations may make it more difficult to encourage cell cycle arrest or death. Despite DNA damage, this failure allows cells to continue dividing. [35]. Furthermore, the Wnt pathway can be excessively triggered as a consequence of APC mutations. KRAs' mutations can trigger the Ras signalling pathway. These triggers can suppress signals that prevent growth, allowing cells to divide and proliferate more easily [36].Genetic and epigenetic changes are the primary cause of adenoma turning into invasive carcinoma. These changes give cancerous cells the ability to invade nearby tissues, travel through the circulation, and spread to other anatomical locations through the metastatic process. [37]. While TP53, APC, and KrAs mutations are commonly seen in adenocarcinomas, including colorectal adenocarcinomas, it is not common for all CRC cases to have mutations in all three genes. [34]. Individual differences in the precise profile of mutations may result from various circumstances, including ulcerative colitis's persistent inflammation, which can hasten the development of cancer.

### 2.7 Gut microbiota and CRC

The growth and course of UC, as well as its ability to produce CRC, are greatly influenced by gut flora. [38,39]. IBD patients and healthy individuals have different gut microbiota arrangements. Currently, this discrepancy is linked to a higher chance of colorectal cancer in individuals with IBD. Dysbiosis, chronic inflammation, metabolite production, the immunological response of the mucosa, genomic instability and DNA damage, and defense by good bacteria are the main factors that contribute to UC-CRC. As was previously mentioned, the gut microbiota plays a vital role in the growth of UC-induced colorectal cancer. Dysbiosis, which is defined by an unbalanced composition of obliging and devious gut microbiota, is allied with pro-inflammatory and carcinogenic mediator production and chronic tissue inflammation, all of which raise colorectal cancer risk. [40]. The protective mucosal barrier may be compromised by dysbiosis, resulting in cancer development and ongoing inflammation. The intestinal mucosal barrier is believed to be compromised by dysbiosis, which is characterized by a rise in particular bacteria like Escherichia coli and enterotoxigenic Bacteroides fragilis (ETBF). More germs can go from the lumen to the inside of the tissue as a result of this breakdown. Chronic tissue inflammation follows, generating carcinogenic and pro-inflammatory molecules that increase the risk of colon cancer. Additionally, dysbiosis promotes intestinal carcinogenesis by activating the cytokines of Type 1 and Type 17 T helper cells. For UC patients, this dysbiosis feedback loop is essential to the advancement of inflammation, dysplasia, and cancer. [41]. Via pathogen-associated molecular patterns (PAMPs), Bacteria can induce an immunological response by communicating, including nucleotide-binding oligomerization domain receptors, Retinoic acid-inducible gene-I-like receptors (RLRs), & Toll-like receptors (TLRs) [42]. The invasion of commensal microorganisms and their component molecules activates TLRs on tumour-infiltrating myeloid cells. This activation occurs because of myeloid differentiation factor 88 (MyD88), which stimulates the synthesis of pro-inflammatory cytokines such as interleukin (IL)-23. IL-23 promotes the production of IL-17A, IL-6, and IL-22 [43]. Acting the NF-κB and STAT3 signalling pathways leads to increased tumour cell proliferation. TLRs and TNF-α can trigger NF-κB, resulting in the transcription of cancer-associated genes like COX-2. As a result, the tumour suppressor p53 pathway induces intestinal epithelium cell loss and the ensuing degradation of the intestinal barrier, which permits bacterial migration. [44]. Additionally, through TLR and MyD88 signalling, symbiotic microbial species and their elements stimulate the production of IL-17C in shifted intestinal epithelial cells (IECs) via an autocrine mechanism. IL-17C promotes Bcl-2 and Bcl-xL communication in IECs, increasing carcinogenesis and tumour cell survival.

## 3 Lupeol and its promising role in therapeutic applications

### 3.1 Sources of lupeol in nature

As a pentacyclic triterpenoid, lupeol is found in many plant species, making it a versatile and easily accessible phytochemical. It is especially abundant in fruits like mangoes (*Mangifera indica*), olives (*Olea europaea*), and guavas (*Psidium guajava*), which are commonly consumed in human diets, as well as vegetables like white cabbage (*Brassica oleracea*). Medicinal plants like Aloe vera, *Crateva adansonii*, leaves of *Alstonia scholaris* [44]*Tamarindus indica* are also rich in lupeol, and their therapeutic properties have been used historically in folk medicine [45].

Lupeol's richness in nutritional plants and medicinal herbs makes it more accessible as a natural cure, while its worldwide availability highlights its importance in traditional medicine systems. *Crateva adansonii*, a traditional Indian medicine herb, contains high quantities of lupeol and has been proven to exhibit anti-inflammatory characteristics. Similarly, Aloe vera has been extensively researched for its wound-healing and antioxidant properties, partly due to its lupeol content [46].

### 3.2 Pharmacokinetics and bioavailability

Despite its vast pharmacological potential, lupeol's medicinal use is limited by its low aqueous solubility and oral bioavailability [47]. This is due to its lipophilicity, which limits absorption and systemic circulation. Once ingested, lupeol is bio-transformed in the liver, predominantly by the cytochrome P450 enzyme system, producing metabolites with potentially improved bioactivity. Furthermore, lupeol has a lengthy half-life, allowing it to exert long-lasting pharmacological effects. However, first-pass metabolism in the liver lowers its systemic concentration, necessitating novel delivery modalities to achieve optimal therapeutic effects. Future studies should combine bioavailability-enhancing approaches with clinical trials to determine their efficacy in humans [48].

To address these constraints, researchers created sophisticated drug delivery devices. Nanoparticle formulations, liposomal encapsulation, and micellar systems have all been investigated to increase lupeol's solubility, stability, and bioavailability. Studies have shown that nano-formulations improve cellular absorption and therapeutic efficacy, particularly in anti-cancer and anti-inflammatory conditions. In preclinical trials, liposome-encapsulated lupeol revealed enhanced bioavailability and tumour-suppressive effects [46].

### 3.3 Safety and toxicity profile

Lupeol is generally safe, with preclinical studies showing no adverse effects even at doses up to 2 g/kg in rodents. Toxicological evaluations revealed no significant changes in hematological, biochemical, or histopathological parameters. While high concentrations of lupeol may induce cytotoxic effects, such as apoptosis or suppressed cell proliferation in vitro, these are absent at clinically relevant doses. However, data on chronic toxicity, reproductive effects, and safety in vulnerable populations remain limited, highlighting the need for further research to support its clinical application [48].

### 3.4 Lupeol's chemical properties

**Fig. 6:**
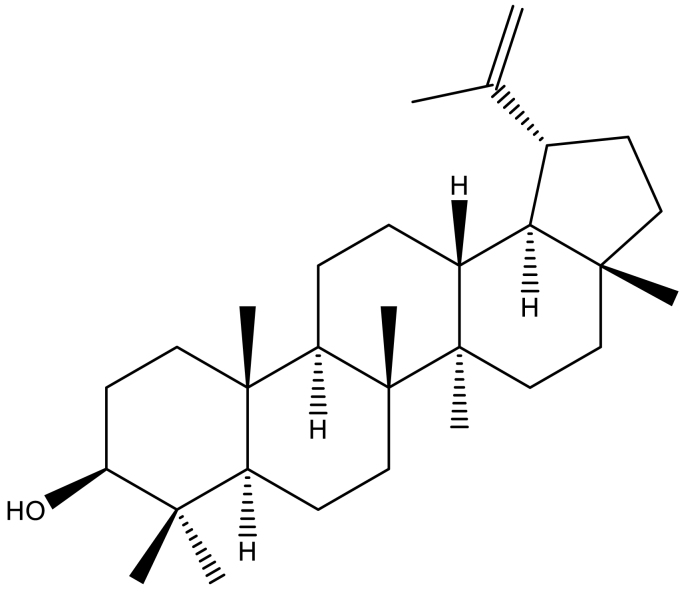
The Chemical structure of lupeol.

A Lupeol[(3-Beta)-Lup-20(29)-en-3-ol] is a kind of triterpene (fig-6), part of a collection of secondary plant metabolites that play a role in environmental interactions, particularly during infections or physical damage [49]. Its biosynthesis, a highly intricate process, is facilitated by triterpene synthases. The pathway has been extensively investigated and can include either five six-membered rings, as seen in ursanes and lanostanes, or four six-membered rings with one five-membered ring, as in lupanes and hopanes. Lupeol, a triterpenoid, has the chemical formula C_30_H_50_O, a molecular weight of 426.7174 g/mol, and a melting point of 215–216 °C. Its infrared spectrum indicates the occurrence of a hydroxyl group and an olefinic moiety at 3235 cm^−1^ and 1640 cm^−1^, respectively. The ^1^H NMR spectrum reveals seven methyl singlets along with an olefinic function, characteristic of triterpenes. HPLC and mass spectrometry (MS) analysis reveal an initial ion peak at *m/z* 409, corresponding to [M + H—18] + [46].

Lupeol, which is often present in over-the-counter medications, has considerable pharmacological actions like antioxidant, anti-inflammatory, and anti-angiogenic properties [50]. It boosts immunological responses to peroxidic oil-induced tumour growth, regulates lipid profiles, and protects against hypercholesterolemia-related stomach damage and immune dysfunction [51].

### 3.5 Mechanistic insights into the colorectal cancer potential of lupeol

#### 3.5.1 Anticancer and apoptotic efficacy

Apoptosis, formerly referred to as programmed cell death, is a developmental phenomenon required for tissue growth and homeostasis. In pathological circumstances such as cancer, cells misplace their capability to undergo apoptosis-induced death, allowing for unrestrained cell division. Cancer cells frequently overexpress numerous critical proteins to prevent the apoptotic cascade from occurring. Overexpression of anti-apoptotic molecules is one strategy cells utilise to avoid programmed cell death. Small molecule inhibitors (SMI) restrict the anti-apoptotic processes of several protein molecules, including B-cell lymphoma-2 (Bcl-2), B-cell lymphoma extra-large (Bcl-xL), etc [54]. The p53 gene, which encodes a 53 KDa nuclear phosphoprotein, originates on the human chromosome's short arm [35,55]. The speed at which breast cancer cells commit apoptosis can be increased by appropriately upregulating the gene mentioned above. The inner and outer membranes of mitochondria may include the apoptotic gene Bcl-2. Cell death can occur when the caspase gene is activated due to low Bcl-2 gene expression. The research conducted found that the proliferation of cancer cells is facilitated by an equilibrium of pro- and anti-apoptotic proteins, including BCL2 and BAX [56]. The cell cycle and tumour are inextricably linked because their machinery controls cell growth. Cancer cells divide continuously, but normal cells divide just a few times before growth stops [57]. Numerous studies have revealed that lupeol can support apoptosis in cancer cells in a variety of ways. The imbalance between pro-apoptotic and anti-apoptotic proteins causes cancer cells to frequently avoid apoptotic procedures [58]. In a variety of cancer cell types, like LNCaP, A431, SMMC7721, HepG2, SW480, HCT116, HEp-2, MCF-7, UPCI: SCC-131, and HeLa cells, lupeol has been demonstrated to trigger the mitochondrial-mediated apoptotic mechanism [32,59]. For example, lupeol treatment may augment the Bax: Bcl-2 ratio, raise reactive oxygen species (ROS) levels, and encourage PARP cleavage. This method can initiate the apoptotic execution phase by activating caspases [60] Numerous research has revealed that lupeol has a high potential for cancer prevention, including liver, lung, bladder, colorectal, and osteosarcoma [55,61] Apoptosis induction, preventing cancer cell migration and invasion, and inhibiting cell proliferation are a few of the biochemical processes at play [[62], [63], [64]].

**Fig. 7:**
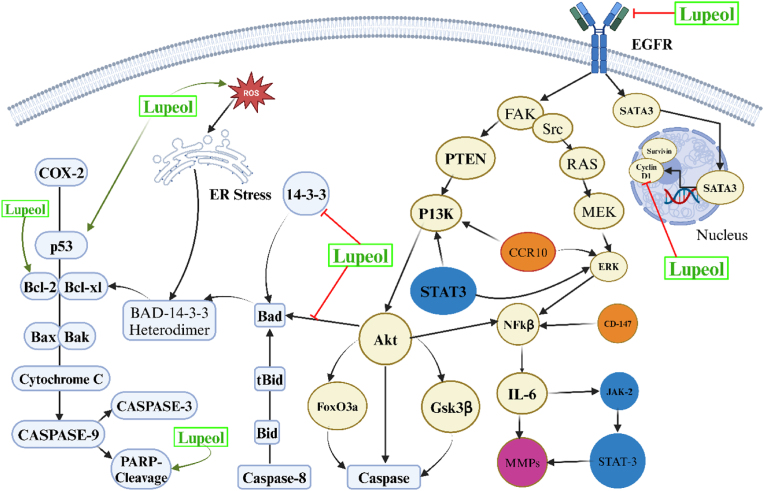
Lupeol's Mechanism on the Apoptotic Pathway. COX-2, Cyclooxygenase-2; p53, Tumor Protein p53; Bcl-2, B-cell Lymphoma 2; Bcl-xl, B-cell Lymphoma Extra Large; Bax, Bcl-2-Associated X Protein; Bak, Bcl-2 Antagonist/Killer; Cytochrome C, Cytochrome Complex C; Caspase-3, Cysteine Aspartate Protease 3; Caspase-8, Cysteine Aspartate Protease 8; Caspase-9, Cysteine Aspartate Protease 9; PARP, Poly (ADP-Ribose) Polymerase; ER Stress, Endoplasmic Reticulum Stress; ROS, Reactive Oxygen Species; BAD, Bcl-2-Associated Death Promoter; 14-3-3, 14-3-3 Protein Family; EGFR, Epidermal Growth Factor Receptor; STAT3, Signal Transducer and Activator of Transcription 3; SATA3, Likely a variant representation of STAT3; PI3K, Phosphoinositide 3-Kinase; PTEN, Phosphatase and Tensin Homolog; FAK, Focal Adhesion Kinase; Src, Proto-Oncogene Tyrosine-Protein Kinase Src; RAS, Rat Sarcoma Virus Protein Family; MEK, Mitogen-Activated Protein Kinase Kinase; ERK, Extracellular Signal-Regulated Kinase; CCR10, C–C Motif Chemokine Receptor 10; NF-κβ, Nuclear Factor Kappa B; IL-6, Interleukin-6; JAK-2, Janus Kinase 2; MMPs, Matrix Metalloproteinases; CD-147, Cluster of Differentiation 147; Akt, Protein Kinase B (PKB); FoxO3a, Forkhead Box O3a; and Gsk3β, Glycogen Synthase Kinase 3 Beta.

Fig. 7 shows how lupeol impacts the apoptosis process. Molecularly targeted drugs have transformed cancer treatment by allowing for the personalized treatment of tumours fueled by specific mutations. These targeted drugs differ from traditional chemotherapies since they are meant to precisely interfere with the activities of certain signalling proteins whose actions are characteristically limited to malignant tissue. Conventional chemotherapy can kill both tumour cells and healthy cells. Nowadays, targeted molecular treatment that uses beneficial monoclonal antibodies or small-molecule medications to interfere with signal transduction pathways is the main focus of accurate medication in the management of cancer. These approaches are currently being utilized as first-line therapies in clinical settings [65,66]. In a similar vein, doxorubicin and lupeol collaborated to decrease tumour growth. Taking lupeol alone or in conjunction with a low dose of doxorubicin and cisplatin resulted in no side effects such as infection, diarrhoea, or weight loss. The lung, heart, tongue, liver, spleen, and kidney were among the typical organs whose hematoxylin and eosin slices showed no signs of necrosis or apparent cell death. Additionally, lupeol significantly reduces tumour size when used in combination with low-dose chemotherapy medications. Compared to chemotherapy drugs alone, lupeol + cisplatin/doxorubicin treatment significantly increased cancer cell-killing [67]. Utilizing the TNBC cell line MDA-MB-231, the combinatorial strategy of lupeol and 5FU was investigated. This technique successfully inhibited wound recovery and proliferation. Additionally, mesenchymal phenotypes and drug resistance are well-known characteristics of MDA-MB-231 cells [68].

## 4 Preclinical and clinical evidence for lupeol in colorectal cancer

Lupeol, a naturally occurring triterpene existing in numerous fruits and medicinal herbs, has received interest for its possible anticancer qualities, particularly in colorectal cancer (CRC). Preclinical studies have studied its efficacy against CRC, focusing on mechanisms such as inhibiting the Wnt/β-catenin signalling pathway, inducing apoptosis, and suppressing cancer stemness (Table 2).

**Table 2:** Preclinical and emerging clinical evidence of lupeol in colorectal cancer.

Study Title	Key Findings	Reference
Lupeol, a dietary terpene, targeted colorectal cancer cells through active Wnt signalling.	Lupeol selectively inhibited CRC cells by active Wnt/β-catenin signalling, reducing cell viability, inducing apoptosis, and decreasing nuclear β-catenin levels. It also downregulated Wnt target genes, suggesting it is probable as a therapeutic agent for CRCs with aberrant Wnt signalling.	[63]
Lupeol disrupts the ER stress-signaling pathway and promotes apoptosis in Oxaliplatin-resistant LoVo colorectal cancer cells by inhibiting ABCG2 expression.	Lupeol-induced apoptosis in resistant to oxaliplatin LoVo CRC cells by modulating the endoplasmic reticulum stress-signaling pathway and downregulating ABCG2 expression, highlighting its potential to overcome chemoresistance in CRC treatment.	[69]
Lupeol suppresses stemming in colon cancer-like cells and reduces tumour formation.	Without influencing cell viability or proliferation, luteol prevented invasion and tumour development in colon cancer stem-like cells, indicating that it may be used to target cancer stemness and stop tumour growth.	[70]
Lupeol triterpene is a new diet-based microtubule-targeted agent.	Lupeol blocked proliferation and migration, triggered apoptosis, and produced cell cycle arrest in CRC cells by targeting microtubules, indicating its potential as a microtubule-targeting agent in CRC therapy.	[71]
Lupeol reduces proliferation and migration in dual colorectal cancer cell lines via suppressing Wnt/β-catenin signalling.	Lupeol suppressed Wnt/β-catenin signalling, reducing progress and migration in CRC cell lines. This supports its effectiveness as a Medicinal Agent targeting this pathway in CRC.	[72]
Lupeol is an innovative blocker of Wnt/β-catenin signalling.	Lupeol subdued Wnt/β-catenin signalling, resulting in decreased invasion and metastasis in CRC cells, suggesting its potential to prevent CRC progression.	[73]

These studies collectively suggest that lupeol holds promise as a therapeutic agent in CRC by targeting key pathways involved in cancer progression and resistance (Table 3). However, clinical trials are obligatory to regulate its effectiveness and safety in humans.

**Table 3:** Patents associated with Lupeol.

S. No.	Patent Title	Patent Number & Year	Country	Ref.
**1**	Lupeol-type triterpene derivatives as antivirals	US9,067,966B2 & 2015	US	[78]
**2**	Lupeol is an anti-tumour agent and uses thereof	US 8,618,082 B2 & 2013	United States Patent	[79]
**3**	Methods of treating antifungal infections using lupeol	US 20040072807A1 & 2004	United States Patent	[80]

## 5 Challenges and future directions

### 5.1 Improving bioavailability and targeted delivery

One of the major challenges in the therapeutic use of lupeol is its deprived bioavailability due to low water solubility and limited systemic absorption. As a lipophilic compound, lupeol faces dissolution and rapid metabolism issues, restricting its therapeutic potential. To address this, the development of nanoparticle-based delivery systems, including liposomes, nanoparticles of polymers, and solid lipid nanoparticles, has revealed the ability to enhance solubility and absorption. Additionally, targeted delivery systems using cancer-specific ligands or monoclonal antibodies can help ensure lupeol reaches tumour tissues effectively while minimising toxicity to healthy cells. Encapsulation of lupeol into micelles, hydrogels, or nanocarriers has also demonstrated improvements in its pharmacokinetics and tumour-targeting capabilities [74,75].

### 5.2 Developing lupeol-based drug formulations

Despite its anticancer potential, lupeol has not yet been formulated into clinically available drug products. Developing standardised lupeol-based formulations remains a critical goal in harnessing its therapeutic benefits. Efforts are underway to design lupeol-loaded formulations, such as nanoemulsions, tablets, and injectable suspensions, which could address issues of stability and bioavailability. Combining lupeol with bioavailability enhancers, such as piperine or lipid carriers, has shown promise in improving systemic absorption. Moreover, integrating lupeol into combination therapies with existing chemotherapeutic agents like 5-fluorouracil (5-FU) can enhance its efficacy, reduce resistance, and lower the toxicity associated with standard treatments [76].

### 5.3 Exploring novel derivatives of lupeol for enhanced activity

The chemical structure of lupeol provides opportunities for the development of novel derivatives with improved anticancer properties, stability, and pharmacokinetics. Recent studies have focused on modifying lupeol's hydroxyl group or triterpenoid scaffold to generate derivatives with enhanced bioactivity and solubility. Novel semi-synthetic lupeol derivatives have demonstrated greater anticancer potential in preclinical models, targeting multiple oncogenic pathways with improved potency [77]. Furthermore, molecular docking and structure-activity relationship (SAR) studies are employed to design derivatives with higher specificity toward colorectal cancer cells. Screening libraries of lupeol analogues, conducting preclinical studies on newly developed derivatives, and utilizing in silico modelling will be critical to identifying lead compounds for CRC therapy [76].

## 6 Conclusion

A major global health concern, colorectal cancer (CRC) is closely connected to chronic inflammation and the deregulation of several signalling pathways, including NF-κB, STAT3, COX-2, and MMPs. Pro-inflammatory mediators like TNF-α, IL-6, and IL-23 promote these pathways, encouraging tumour growth, angiogenesis, and metastasis. Addressing these mechanisms is critical in mitigating CRC development, particularly in conditions such as ulcerative colitis (UC)-associated CRC. Lupeol, a quite naturally pentacyclic triterpenoid, has emerged as a promising therapeutic candidate due to its extensive pharmacological profile, encompassing antioxidant, anti-inflammatory, and anticancer properties. Despite its immense potential, lupeol's deprived aquatic solubility and bioavailability have posed challenges for its clinical application. However, advancements in nano-based delivery systems and the progress of lupeol derivatives have successfully enhanced its bioavailability and amplified its therapeutic efficacy. This review underscores the multifaceted role of lupeol in modulating CRC-related signalling pathways and its potential as a nutraceutical intervention for CRC. Furthermore, it highlights the need for continued research to optimize the utilization of lupeol, paving the way for its development as a novel pharmaceutical agent. By consolidating current knowledge, this review aims to inspire further exploration into the healing applications of lupeol and its derivatives, offering new avenues for effective CRC management.

## Author contribution

Mohd Asad Farooqi: Wrote and figure preparation, Review, Investigation, Edit, Data curation.

Kantrol Kumar Sahu: Supervision, Corrected the paper draft, Project administration, Conceptualization.

## Declaration of generative AI in scientific writing

The authors declare no AI generated work in this paper.

## Funding sources

Not funded from anywhere.

## Conflict of interest

The authors declare that they have no known competing financial interests or personal relationships that could have appeared to influence the work reported in this article.
